# Accelerating Ice Loss From Peripheral Glaciers in North Greenland

**DOI:** 10.1029/2022GL098915

**Published:** 2022-06-16

**Authors:** Shfaqat A. Khan, William Colgan, Thomas A. Neumann, Michiel R. van den Broeke, Kelly M. Brunt, Brice Noël, Jonathan L. Bamber, Javed Hassan, Anders A. Bjørk

**Affiliations:** ^1^ DTU Space Technical University of Denmark Kongens Lyngby Denmark; ^2^ Department of Glaciology and Climate Geological Survey of Denmark and Greenland Copenhagen Denmark; ^3^ NASA Goddard Space Flight Center Greenbelt MD USA; ^4^ Institute for Marine and Atmospheric Research Utrecht Utrecht University Utrecht The Netherlands; ^5^ Earth System ScienceInterdisciplinary Center University of Maryland College Park MD USA; ^6^ Bristol Glaciology Centre University of Bristol Bristol UK; ^7^ Department of Aerospace and Geodesy Data Science in Earth Observation Technical University of Munich Munich Germany; ^8^ Department of Geosciences and Natural Resources University of Copenhagen Copenhagen Denmark

**Keywords:** Greenland, ice mass loss, Icesat‐2, peripheral glacier, satellite altimetry, sea level rise

## Abstract

In recent decades, Greenland's peripheral glaciers have experienced large‐scale mass loss, resulting in a substantial contribution to sea level rise. While their total area of Greenland ice cover is relatively small (4%), their mass loss is disproportionally large compared to the Greenland ice sheet. Satellite altimetry from Ice, Cloud, and land Elevation Satellite (ICESat) and ICESat‐2 shows that mass loss from Greenland's peripheral glaciers increased from 27.2 ± 6.2 Gt/yr (February 2003–October 2009) to 42.3 ± 6.2 Gt/yr (October 2018–December 2021). These relatively small glaciers now constitute 11 ± 2% of Greenland's ice loss and contribute to global sea level rise. In the period October 2018–December 2021, mass loss increased by a factor of four for peripheral glaciers in North Greenland. While peripheral glacier mass loss is widespread, we also observe a complex regional pattern where increases in precipitation at high altitudes have partially counteracted increases in melt at low altitude.

## Key Points

- Greenland peripheral glacier mass loss increased from 27.2 Gt/yr (February 2003–October 2009) to 42.3 Gt/yr (October 2018–December 2021
- Mass loss increased by a factor of four for peripheral glaciers in North Greenland during February 2003–December 2021
- Enhanced precipitation in northeast Greenland during October 2018–December 2021 resulted in ice thickening at higher elevations

## 1 Introduction

Greenland's peripheral glaciers are an important, but often not separately considered, element of the global sea level rise budget. Ice loss estimates from satellite gravimetry, which cannot separate the peripheral glaciers from the contiguous ice sheet, are often blended with altimetry and mass‐budget ice loss estimates that only sample the contiguous ice sheet. Satellite gravimetry‐based community assessments of Greenland's recent sea level rise contribution note that they effectively employ “Greenland ice loss” as being synonymous with “Greenland ice sheet ice loss” (IMBIE Team, 2020; Shepherd et al., 2012).

While peripheral glaciers comprise 4% of Greenland's ice‐covered area, their specific ice loss is disproportionately high in comparison to that of the ice sheet (11 ± 2%, as we describe here). The most recent laser altimetry estimates of Greenland peripheral glacier ice loss pertain to the 2003–2009 Ice, Cloud, and land Elevation Satellite (ICESat) observational period. These estimates range between 28 ± 12 and 44 ± 18 Gt/yr of ice loss, depending on which glaciers are classified as “peripheral” (Bolch et al., 2013; Gardner et al., 2013). This is comparable to the ice loss from Ellesmere Island, adjacent to northeast Greenland (Sasgen et al., 2022; Wouters et al., 2019). Peripheral glaciers were responsible for >10% of the Greenland ice loss observed during this period (Colgan et al., 2013; Gardner et al., 2013).

Regional climate modeling suggests that increasing air temperatures and meltwater percolation have contributed to a recent, and sharp, decrease in firn pore volume across Greenland's peripheral glaciers (Noël et al., 2017). The ability of peripheral glaciers to buffer their response to climate change, by retaining meltwater via refreezing in porous firn, is expected to decrease with this decline in firn pore volume, promoting acceleration of mass loss (van Angelen et al., 2013). Here, we provide the first laser altimetry assessment of changing ice loss rates from Greenland peripheral glaciers that bridges both the ICESat and ICESat‐2 periods of February 2003–December 2021.

## 2 Data and Methods

### 2.1 Elevation Changes During February 2003–October 2009 From ICESat

We use ICESat data from February 2003 to October 2009 (Schenk & Csatho, 2012; Smith et al., 2020; Zwally et al., 2014) to estimate elevation changes over the ice surface. We estimate height changes over the ice surface on a regular grid with a resolution of 500 × 500 m that covers all of Greenland's peripheral glaciers. We use all available ICESat data to create height time series at each grid point. At each grid point, we fit a trend, a second‐order surface topography, and a seasonal term to account for the annual surface changes (Khan et al., 2022; Schenk & Csatho, 2012; Sørensen et al., 2011) (see Supporting Information S1 for details).

The observed height change rates from ICESat are spatially interpolated into a regular grid of 500 × 500 m. The interpolation is performed using the ordinary kriging method (Hurkmans et al., 2014; Nielsen et al., 2013). We use the observed elevation change rates to estimate an empirical semivariogram. Next, we fit an exponential model variogram (with a range of 60 km) to the empirical semivariogram to take the spatial correlation of elevation change rates into account in the error budget. For each grid point, we estimate elevation change rate dh_i,krig,_ and associated uncertainty error.

We correct the observed height change rates for firn compaction, elastic uplift rates from present‐day mass changes, and long‐term past ice changes (Glacial Isostatic Adjustment—GIA). We correct for GIA using the GNET‐GIA empirical model of Khan et al. (2016). For each grid point, we estimate the GIA uplift rate dh_GIA_ and the associated uncertainty s_GIA_. To correct for elastic uplift of the bedrock, we convolve ice loss estimates of peripheral glaciers and the Greenland ice sheet with Green's functions derived by Wang et al. (2012) for elastic Earth model iasp91 (Wang et al., 2012) with a refined crustal structure from Crust 2.0 (Laske et al., 2012).

### 2.2 Elevation Changes During October 2018–December 2021 From ICESat‐2

We estimated elevation changes for Greenland's peripheral glaciers using ICESat‐2 data from October 2018 to December 2021. We use ICESat‐2 Algorithm Theoretical Basis Document for Land Ice Height (ATL06) Release 004 retrieved from https://nsidc.org/data/atl06 (Smith et al., 2021). We estimate elevation changes using the same method as described for ICESat data in the previous section. We also apply the same corrections for elastic uplift, GIA, and firn compaction.

### 2.3 Elevation Changes During October 2008–April 2019

We fill the gap between ICESat and ICESat‐2 satellite missions by merging elevations from both missions. We estimate elevation changes for Greenland's peripheral glaciers by performing a crossover analysis between ICESat and ICESat‐2 data. We use all available ICESat data points from October 2008 to October 2009 and ICESat‐2 data points from October 2018 to April 2019. Our elevation changes of dh point measurements span between 9.0 and 10.5 years. We estimate elevation change rates based on dh differences and apply corrections for elastic uplift, GIA, and firn compaction (see Supporting Information S1).

## 3 Results

### 3.1 Elevation Changes and Ice Loss

Figure 2 shows elevation change rates during February 2003–October 2009, October 2008–April 2019, and October 2018–December 2021.

**Figure 1:**
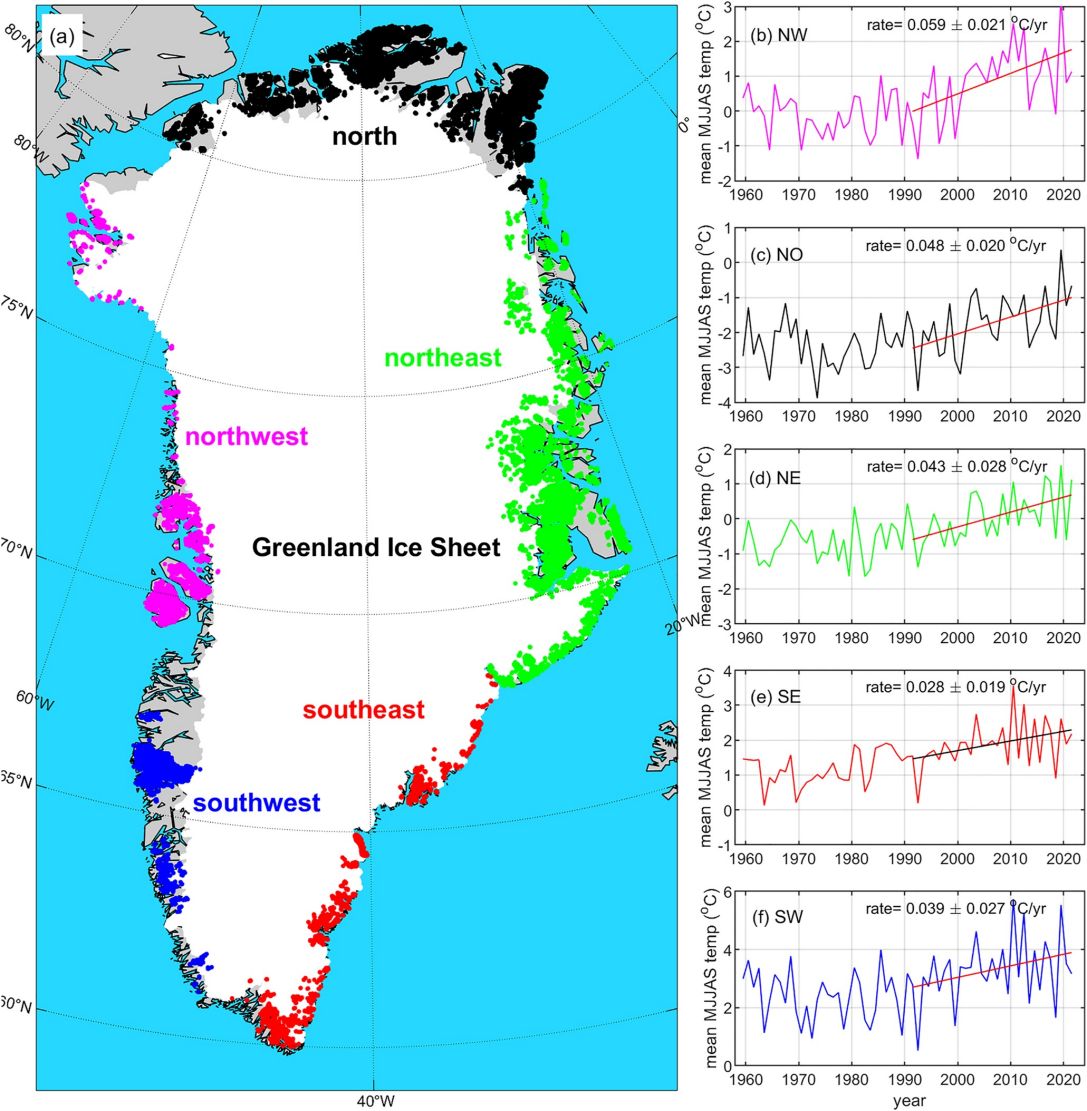
(a) Map of ice‐covered areas in Greenland. Greenland ice sheet (white). Peripheral glaciers in the north (black dots), northeast (green dots), southeast (red dots), southwest (blue dots), and northwest (purple dots). Mean surface air temperature in °C during May–September in (b) the north, (c) northwest, (d) southeast, (e) southwest, and (f) northwest Greenland from RACMO2.3p2. The straight line in panel (b–f) denotes 1990–2021 trend with the rate listed above the line. Area size in km^2^ for each peripheral glacier region and the Greenland ice sheet are listed in Table 1.

**Figure 2:**
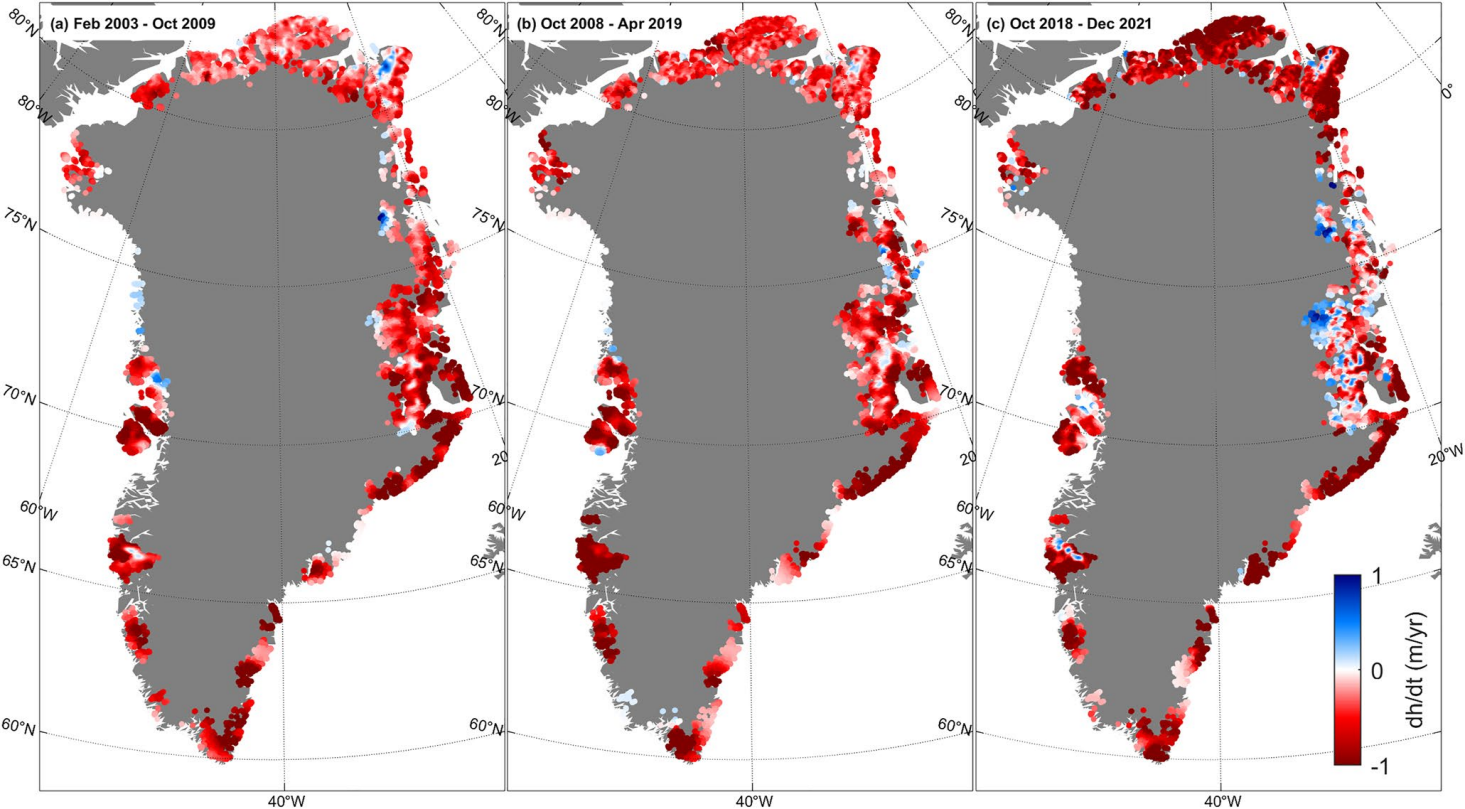
Elevation change rates, in m/yr, during (a) February 2003–October 2009, (b) October 2008–April 2019, and (c) October 2018–December 2021.

Table 1 shows mass change for each of the five peripheral glacier regions considered in this study, along with the contemporaneous mass change of the Greenland ice sheet. The Greenland ice sheet mass change during February 2003–October 2009 was retrieved from Khan et al. (2016), and the mass change during October 2008–April 2019 was retrieved from Khan et al. (2022). For the period from October 2018 to December 2021, we used ICESat‐2 data.

**Table 1:** Peripheral Glacier (This Study) and Ice Sheet (Khan et al., 2016, 2022) Mass Change (Including Correction for Firn, Elastic, and GIA)

Region	Area [km^2^]	Mass change [Gt/yr]	Mass change [Gt/yr]	Mass change [Gt/yr]
		February 2003–October 2009	October 2008–April 2019	October 2018–December 2021
North	35,003	−6.7 ± 1.6	−11.7 ± 1.4	−26.1 ± 1.4
Northeast	21,943	−11.8 ± 2.2	−9.4 ± 2.7	−6.4 ± 2.3
Southeast	2,610	−2.3 ± 0.6	−1.6 ± 1.0	−2.6 ± 0.8
Southwest	7,011	−2.9 ± 0.9	−4.9 ± 0.9	−3.0 ± 0.9
Northwest	5,807	−3.5 ± 0.9	−6.9 ± 0.9	−4.2 ± 0.8
**All peripheral glaciers**	**72,374**	**−27.2 ± 6.2**	**−34.5 ± 6.9**	**−42.3 ± 6.2**
Greenland ice sheet	1,732,859	−218.1 ± 20.1	−258.6 ± 15.6	−262.3 ± 39.0
**All Greenland**	**1,805,233**	**−245.3 ± 26.3**	**−293.1 ± 22.4**	**−304.6 ± 45.2**

*Note.* Bold text indicate total Area or Mass Change.

### 3.2 Complex Regional Ice Loss Patterns

We find large differences in the ice loss trends across the five peripheral glacier regions that we survey. Three regions—southeast, southwest, and northwest—each lost between 1.6 and 6.9 Gt/yr of ice mass. Though they show large temporal variability, their total contribution to ice loss is relatively small. Conversely, the north and northeast regions show striking features of a large and persistent thinning pattern.

**Figure 3:**
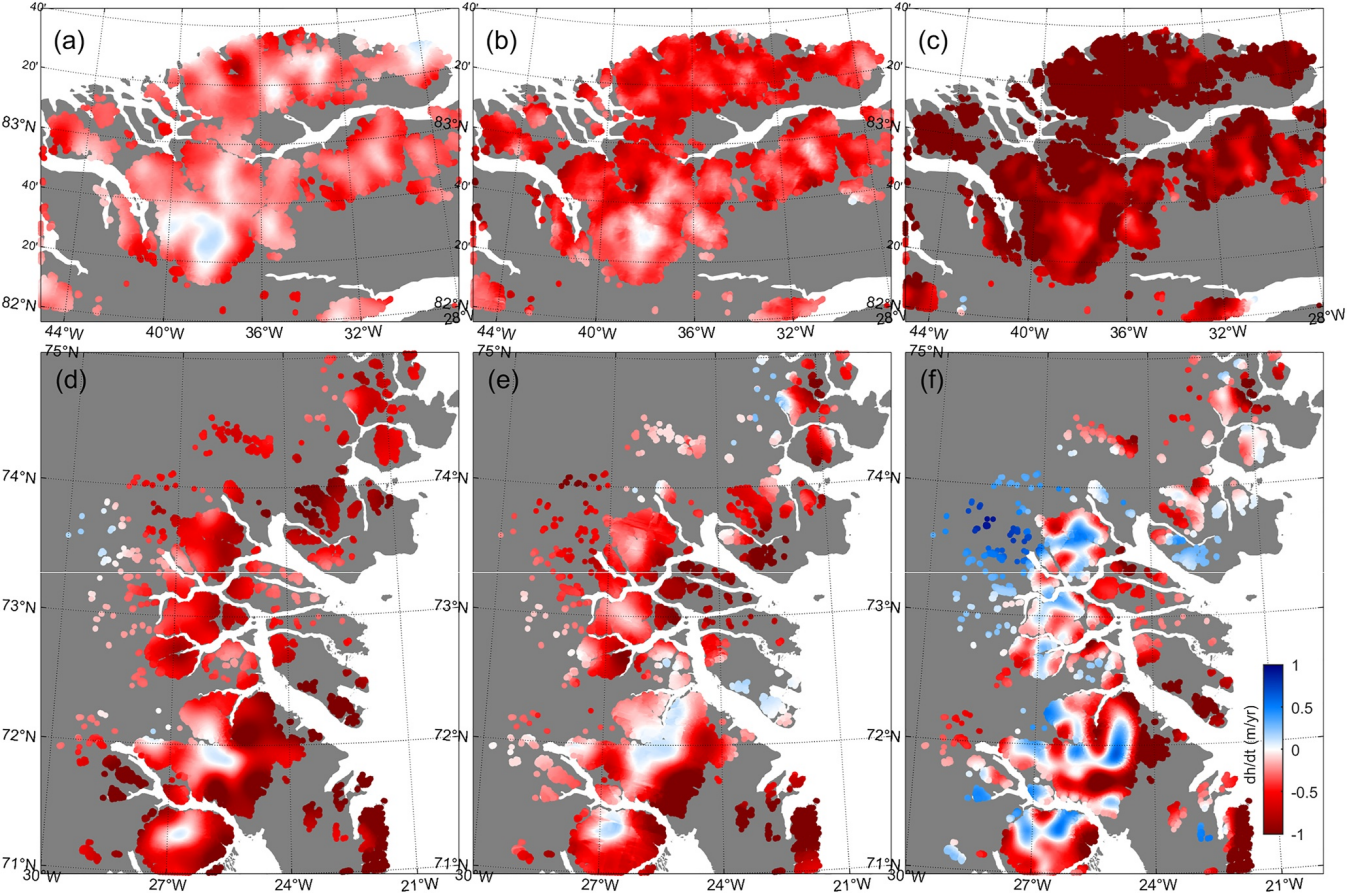
Top row: Detailed elevation change rates of Figure 2 during (a) February 2003–October 2009, (b) October 2008–April 2019, and (c) October 2018–December 2021 for peripheral glaciers in the North. Bottom row: Detailed elevation change rates for peripheral glaciers in the northeast during (d) February 2003–October 2009, (e) October 2008–April 2019, and (f) October 2018–December 2021.

In North Greenland, we observe an increase in the thinning rate during February 2003–December 2021 (Figures 3a–3c), resulting in an increased mass loss rate from 6.7 ± 1.6 Gt/yr during February 2003–October 2009 to 11.7 ± 1.4 Gt/yr during October 2008–April 2019 and finally 26.1 ± 1.4 during October 2018–December 2021. Figures 3a–3c suggests that thinning is spreading to higher elevations over this time. While the highest elevations slightly thickened during February 2003–October 2009, these areas transitioned into slight thinning during October 2008–April 2019 followed by more extensive thinning during October 2018–December 2021.

In northeast Greenland, by contrast, thickening at higher elevations has become more extensive, while lower altitudes have continued to thin (Figures 3d–3f). As a result, the total mass loss rate of the northeastern sector declined from 11.8 ± 2.2 Gt/yr (February 2003–October 2009) to 9.4 ± 2.7 Gt/yr (October 2008–April 2019) and finally 6.4 ± 2.3 (October 2018–December 2021) (see Table 1). We note that the peripheral glacier plateaus in the North sector have a maximum altitude of about 1,000–1,200 m, while the glacier plateaus in the northeast sector have a maximum altitude of about 2,000–3,000 m. Plateau geometry likely plays a key role in sustaining a viable accumulation area.

## 4 Discussion

### 4.1 Disproportional Sea Level Rise Contribution

Previous laser altimetry assessments of peripheral glacier ice loss have excluded the ice sheet and did not use ICESat‐2 data to assess the most recent changes (Bolch et al., 2013; Gardner et al., 2013; Noël et al., 2017). By combining our peripheral glacier assessment with an ice sheet assessment using an analogous altimetry approach and ice mask (Khan et al., 2016, 2022), we present the first consistent, geodetic estimate of ice loss from all of Greenland's land ice derived from laser altimetry. By unambiguously identifying all of Greenland's land ice as either peripheral glacier or ice sheet (Pedersen et al., 2013), we resolve previous ambiguity associated with pairing independent peripheral glacier and ice sheet assessments (Bamber et al., 2018). The peripheral glacier area of the Pedersen et al. (2013) ice mask that we use (72,374 km^2^) is ∼18% smaller than the 88,083 km^2^ of Citterio and Ahlstrøm (2013) ice mask and ∼20% smaller than the 89,720 km^2^ ice mask of Rastner et al. (2012). Our assessment therefore represents a lower bound of peripheral glacier mass loss.

We assess the peripheral glacier contribution to Greenland ice loss as 11 ± 2% during both the February 2003–October 2009 and October 2008–April 2019 periods. During the October 2018–December 2021 period, however, the peripheral glacier contribution to Greenland ice loss increased sharply to 14 ± 2%. This recent increase in the peripheral glacier contribution to Greenland ice loss is associated with a sharp increase in ice loss from North Greenland glaciers (discussed below). Given that Greenland's peripheral glaciers comprise 4% of land ice by area (Pedersen et al., 2013) but are responsible for 14 ± 2% of current Greenland ice loss, their sea level contribution is clearly disproportionately large. This is consistent with the expectation that smaller local glaciers with hypsometry peaking at lower elevations respond more rapidly to climate changes than a larger ice sheet with an extensive and highly elevated interior (Bahr et al., 1998; Noël et al., 2017, 2020).

### 4.2 Accelerated Ice Loss in North Greenland

Peripheral glacier mass loss in North Greenland has effectively quadrupled over the observational period, from 6.7 ± 1.6 Gt/yr during the February 2003–October 2009 ICESat period to 26.1 ± 1.4 Gt/yr during the October 2018–December 2021 ICESat‐2 period. The large mass loss rate during the ICESat‐2 period is strongly influenced by the major melt event in 2019 (Sasgen et al., 2020), followed by relative warm summers in 2020 and 2021 (see Figure 1c). North Greenland is now responsible for ∼60% of all Greenland peripheral glacier mass loss—more than all other regions combined.

Figures 1b–1f shows regional mean surface air temperatures in °C during May–September (MJJAS) from the regional climate model RACMO2.3p2 (Noël et al., 2018). The mean air temperatures are estimates over peripheral glacier areas highlighted in Figure 1a. Since c. 1990, north, northeast, and northwest Greenland have been experiencing a greater rate of warming than South Greenland (Figures 1b–1f). We estimate an average air temperature trend of 0.048 ± 0.020°C/yr for North Greenland and 0.028 ± 0.019°C/yr for southeast Greenland (see Figure 1). This is attributable to large‐scale and persistent changes in atmospheric circulation, which have resulted in the North Greenland ice sheet ablation area expanding about twice as fast as the South Greenland ablation area (Noël et al., 2019).

The North Greenland peripheral glacier mass loss during the October 2018–December 2021 period is clearly influenced by the extreme 2019 melt season. This extreme summer melt appears to have been caused by two compounding factors: relatively low winter snowfall, which preconditioned ice for an earlier and longer melt season, and relatively stable summer high‐pressure systems, with enhanced melting through increased northward advection of midlatitude air masses (Hanna et al., 2016; Sasgen et al., 2020). As climate change is expected to both lengthen the melt season and enhance midlatitude atmospheric inflow to the Arctic (Cai et al., 2018; Overland et al., 2019; Sasgen et al., 2022), the extreme glacier loss that we observe in North Greenland in 2019 may be considered symptomatic of a future warmer Arctic.

## 5 Conclusions

We present the first comprehensive laser altimetry‐based assessment of ice loss from all of Greenland's land ice, including both peripheral glaciers and the Greenland ice sheet, spanning the period February 2003–December 2021. While peripheral glaciers comprise only a small portion of Greenland's land ice area, mass loss is increasing faster in relation to the ice sheet. In North Greenland especially, peripheral glacier mass loss has increased fourfold over the satellite observation period. In recent years, this area of glaciers has contributed to approximately 10% (Table 1, bold entries) of the total Greenland ice loss.

Greenland's peripheral glaciers contain an estimated total ice volume of 4.7 ± 0.7 cm of sea level equivalent (Huss & Farinotti, 2012). This is comparable to the 4.6 ± 1.5 cm of sea level equivalent for Alaska and Western Canada combined (Millan et al., 2022). Numerical projections suggest that Greenland's peripheral glaciers will not reach peak specific mass loss within the 21st century. Greenland's peripheral glaciers are therefore poised to remain a major player in Greenland's ice loss budget for decades to come. This provides strong motivation to ensure explicit representation of peripheral glacier mass loss in community assessments of Greenland ice loss (IMBIE Team, 2020).

The trends and variability in Greenland's peripheral glacier mass loss that we document are clearly complex in both space and time. Better scientific understanding of the presatellite era mass balance of Greenland's peripheral glacier population would place contemporary mass loss rates into a better temporal context. At present, geodetic estimates of Greenland's peripheral glacier mass balance are a missing component of the 20th century global sea level budget.

## Supporting information

Supporting Information S1

## Data Availability

The Ice, Cloud, and land Elevation Satellite (ICESat) data are available at: https://nsidc.org/data/icesat/data.html. The Ice, Cloud, and land Elevation Satellite‐2 (ICESat‐2) data are available at: https://nsidc.org/data/icesat-2. Surface elevation change rates, firn compaction rates, elastic uplift rates from present‐day mass changes, GIA vertical Land Motion, and air temperature time series are available at the following data repository: https://datadryad.org/stash/share/q3qZmdNu3RSZMmaxjtKQMStLrp9TFFASELjUPxAn3Hw.
