# Supplementary material for: Accelerating Ice Loss From Peripheral Glaciers in North Greenland
Source: Geophys Res Lett. 2022 Jun 16;49(12):e2022GL098915. doi: 10.1029/2022GL098915 (PMC9286807; doi:10.1029/2022GL098915)
Supplement: Supplementary file 1 — Supporting Information S1 [file GRL-49-0-s001.docx]

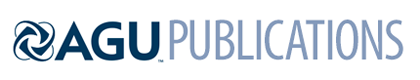


*[Geophysical Research Letters]*

Supporting Information for

**[****Accelerating ice loss from peripheral glaciers in North Greenland]**

[Shfaqat A. Khan*^1^, William Colgan^2^, Thomas A. Neumann^3^, and Michiel van den Broeke^4^, Kelly M Brunt^3^, Brice Noël^4^, Jonathan Bamber^5^, Javed Hassan^1^ and Anders A Bjørk^5^]

[^1^DTU Space, Technical University of Denmark, Denmark

^2^Department of Glaciology and Climate, Geological Survey of Denmark and Greenland, Denmark.

^3^NASA Goddard Space Flight Center, Greenbelt, MD, USA

^4^Institute for Marine and Atmospheric Research Utrecht, Utrecht University, Utrecht, The Netherlands.

^5^Department of Geosciences and Natural Resources, University of Copenhagen, Denmark]

**Contents of this file**

Elevation change rates from ICESat-2 data from October 2018 to December 2021

Elevation changes during February 2003 – October 2009

Elevation changes during October 2008 – April 2019

Correction for glacial isostatic adjustment, elastic uplift, and firn compaction

Table S1: corrections for firn compaction

Table S2: correction for elastic uplift

Table S3: Correction for GIA

Figure S1: Elevation change rates during October 2018 – December 2021

Figure S2: Elevation change rates during October 2018 – December at Flade Isblink.

Figure S3: Elevation change rates as a function of elevation during October 2018 – December.

Figure S4: Elevation change rates during February 2003 – October 2009

Figure S5: Elevation change rates as a function of elevation during February 2003 – October 2009.

Figure S6: Elevation change rates during October 2008 – April 2019

Figure S7: Elevation change rates as a function of elevation during October 2008 – April 2019.

**Elevation change rates from ICESat-2 data from October 2018 to December 2021**

To estimate elevation changes over the ice surface, we use a regular grid with a resolution of 500x500 km that covers Greenland’s peripheral glaciers. We denote the center of each grid point with **C**(*x_0_*, *y_0_*). For each grid point, we select ICESat-2 data with coordinates **P**(*x_i_*, *y_i_*), with a maximum distance of 500 m from **C**. The CryoSat-2 data points with coordinates **P**(*x_i_*, *y_i_*), have elevation *h_i_* measured at time *t_i_*. The index *i* denote the *i*‘th data point.

Here, we use all available ICESat-2 data measured between October 2018 to December 2021 to create surface elevation time series at each grid point **C.** At each grid point C, we fit a trend and a second-order surface topography and a seasonal term to account for the annual surface changes.

For each grid point with center (*x_0_*, *y_0_*), we find the nearest data within 500 m (x_i_, y_i_, h_i_, t_i_) and fit a trend ${H(t_{i})}_{trend}$, a 2-order surface topography $H_{topo}$ and an annual term ${H(t_{i})}_{Annual}$:

$$H(t_{i})={H(t_{i})}_{trend}+H_{topo}+{H(t_{i})}_{Annual}$$

Where ${H(t_{i})}_{trend}$ is,

$${H(t_{i})}_{trend}=a_{1}+a_{2}t_{i}$$

Where $t_{i}$ is the time when the i’th measurement was observed. For simplicity, we used 1 January 2000 as reference time t=0. $a_{1}$to $a_{2}$ are parameters. The parameter $a_{1}$ represents surface elevation at time 1 January 2000. We assume the shape of the surface remains constant during the time interval considered in this study. While many studies use a planer surface (Sørensen et al., 2011; Hurkmans et al., 2014), here, we fit a 2-order polynomial to describe a surface topography of the area of 0.5x0.5 km (Csatho et al., 2014),

$$H_{topo}=a_{3}x+a_{4}y+a_{5}x^{2}+a_{6}y^{2}+a_{7}xy$$

$a_{3}$ to $a_{7}$ are parameters that describe the slope and roughness of the surface. $x$ and $y$ are coordinates of the ICESat-2 data point, but in a system with *x_0_* and *y_0_* as the center. i.e. $x= x_{i}-x_{o}$ and $y= y_{i}-y_{o}$. The annual term is given by,

$${H(t_{i})}_{Annual}=a_{8}cos(t_{i}-a_{9})$$

For each grid point, we create a time series and use least squares adjustment to simultaneously estimate parameters *a_1_* to *a_9_*. Our procedure for deriving ice surface elevation changes is very similar to the method used by, for example, Schenk et al. (2014).

Figure S1(a) shows elevation change rates. We removed local dh/dt outliers using planar regression in 25 km bins for Greenland. Values that fell outside of 10-sigma were removed.

We interpolate elevation change rates onto a regular grid of 0.5x0.5 km. The interpolation was performed using the ordinary kriging method (Hurkmans et al., 2014; Nielsen et al., 2013), except for the area marked as southeast and southwest Greenland in figure S1a. Figure S1(b) shows interpolated elevation change rates and figure S1(c) shows uncertainty associated with elevation changes rates. Figure S2 shows elevation change rates over Flade Isblink located at the northeastern corner of Greenland. Figure S2 is a zoom-in over the are area marked by a square in figure S1(a).


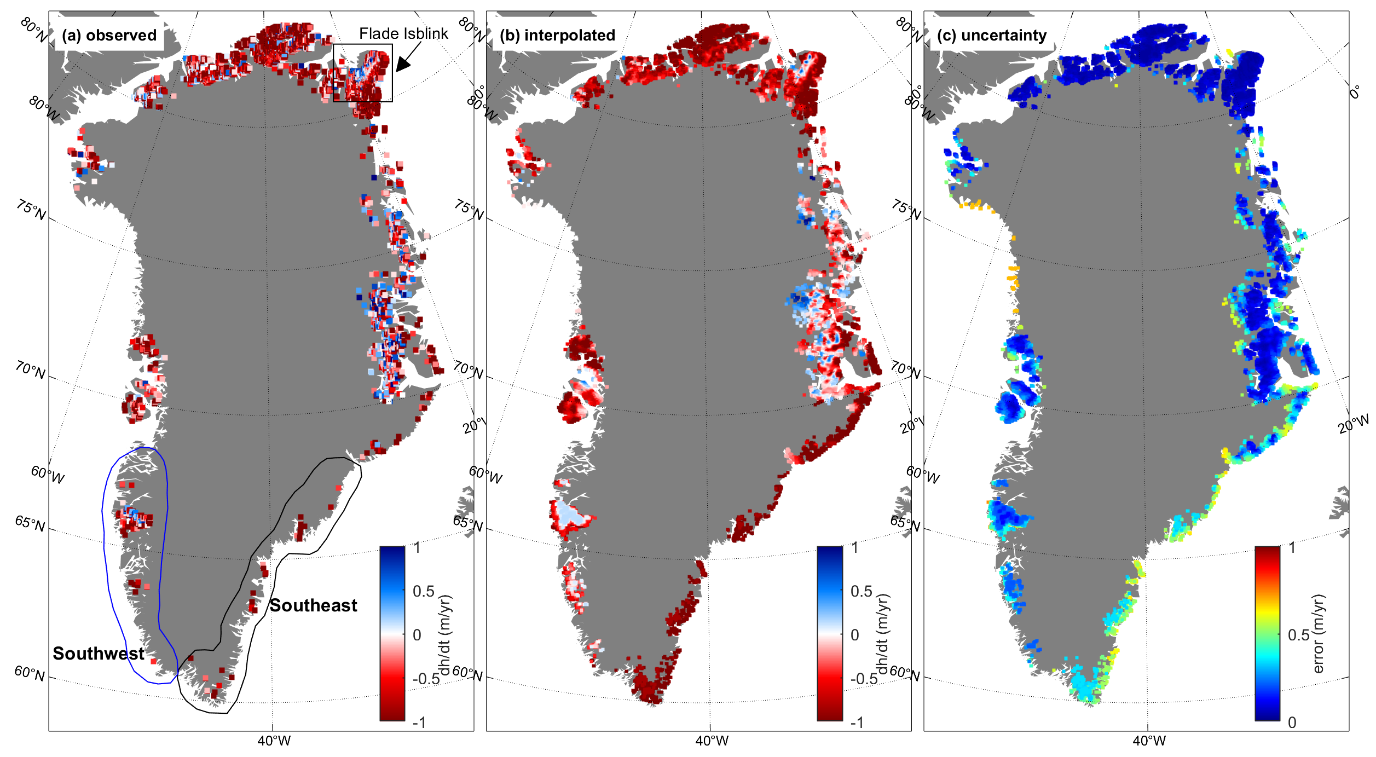


Figure S1. (a) Observed elevation change rates in m/yr during Oct 2018 – Dec 2021 using ICESat-2 data. The black curve marks southeast Greenland and the blue curve marks southeast Greenland. The box in northeast Greenland marks the region shown in figure S2. (b) interpolated elevation change rates and (c) uncertainties of interpolated elevation change rates.


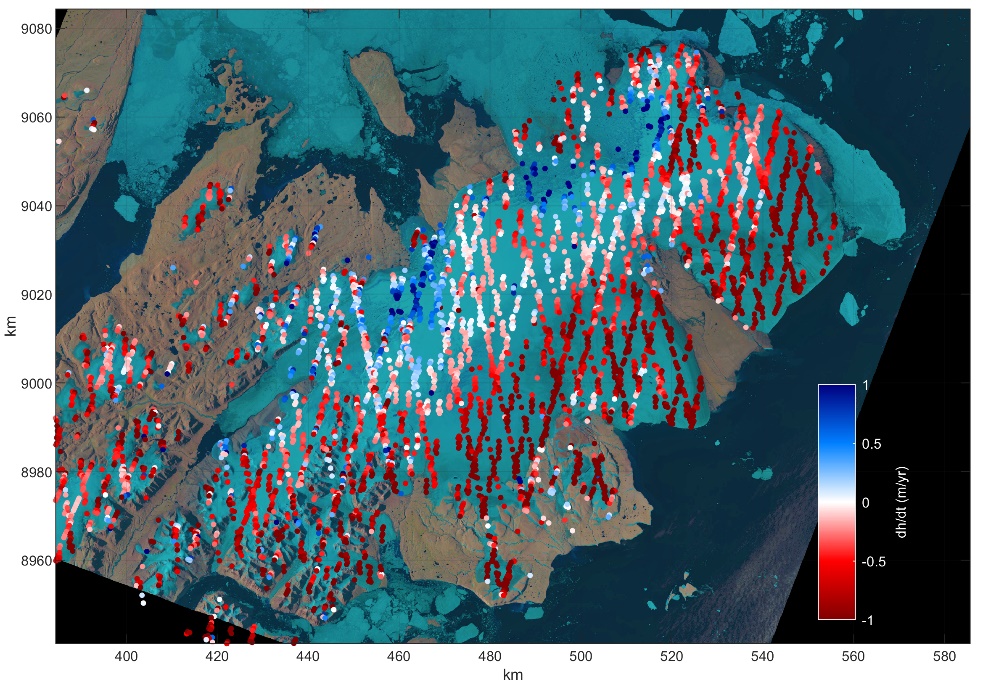


Figure S2. Mean elevation change rates during Oct 2018 – Dec 2021 in m/yr. The figure is a zoom-in on the area marked by a square in figure S1a.

For the two areas, southeast and southwest Greenland we interpolate into a regular grid of 0.5x0.5 km using the method described by Garner et al. (2013). At lower latitudes, e.g. in southeast and southwest Greenland, the amount of ICESat-2 data points are sparse. Therefore, kriging is not recommended, instead, we parameterize dh/dt as a function of elevation within a sub-region as defined in figure S1a. Figure S3 shows elevation change as a function of elevation for southeast and southwest Greenland. We use these functions to estimate the sub-region elevation change rates which are shown in figure S1b.


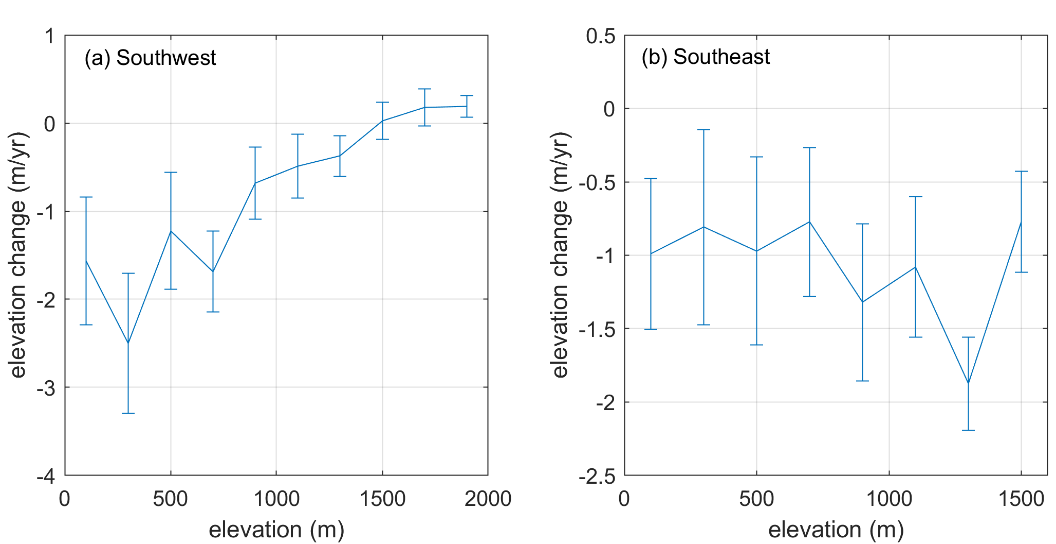


Figure S3. Elevation change rates (dh/dt) and standard deviations within 200 m elevation intervals for glaciers in (a) southwest Greenland and (b) southeast Greenland during Oct 2018 – Dec 2021 using ICESat-2 data.

**Elevation changes during February 2003 – October 2009**

We estimated elevation changes for Greenland’s peripheral glaciers using ICESat data from February 2003 to October 2009. We estimate elevation changes using the same method as described in the previous section.

**
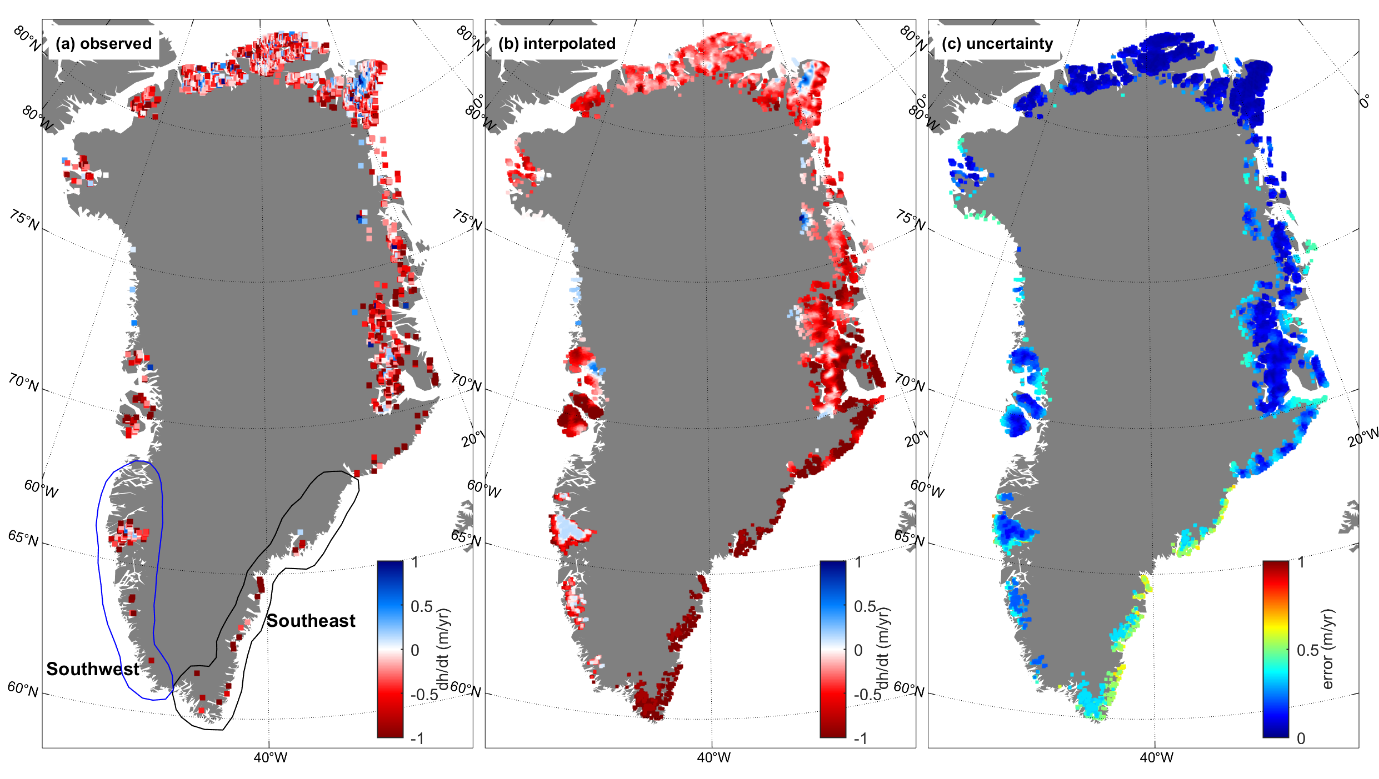
**

Figure S4. (a) Observed elevation change rates in m/yr during Feb 2003 – Oct 2009 using ICESat data. The black curve marks southeast Greenland and the blue curve marks southeast Greenland. (b) interpolated elevation change rates and (c) uncertainties of interpolated elevation change rates.

**
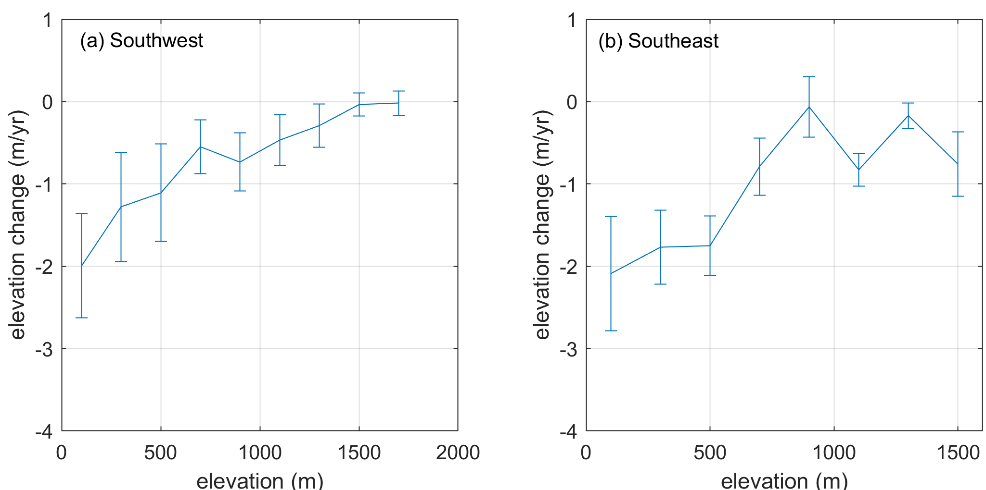
**

Figure S5. Elevation change rates (dh/dt) and standard deviations within 200 m elevation intervals for glaciers in (a) southwest Greenland and (b) southeast Greenland during Feb 2003 – Dec 2009 using ICESat data.

**Elevation changes during October 2008 – April 2019**

**
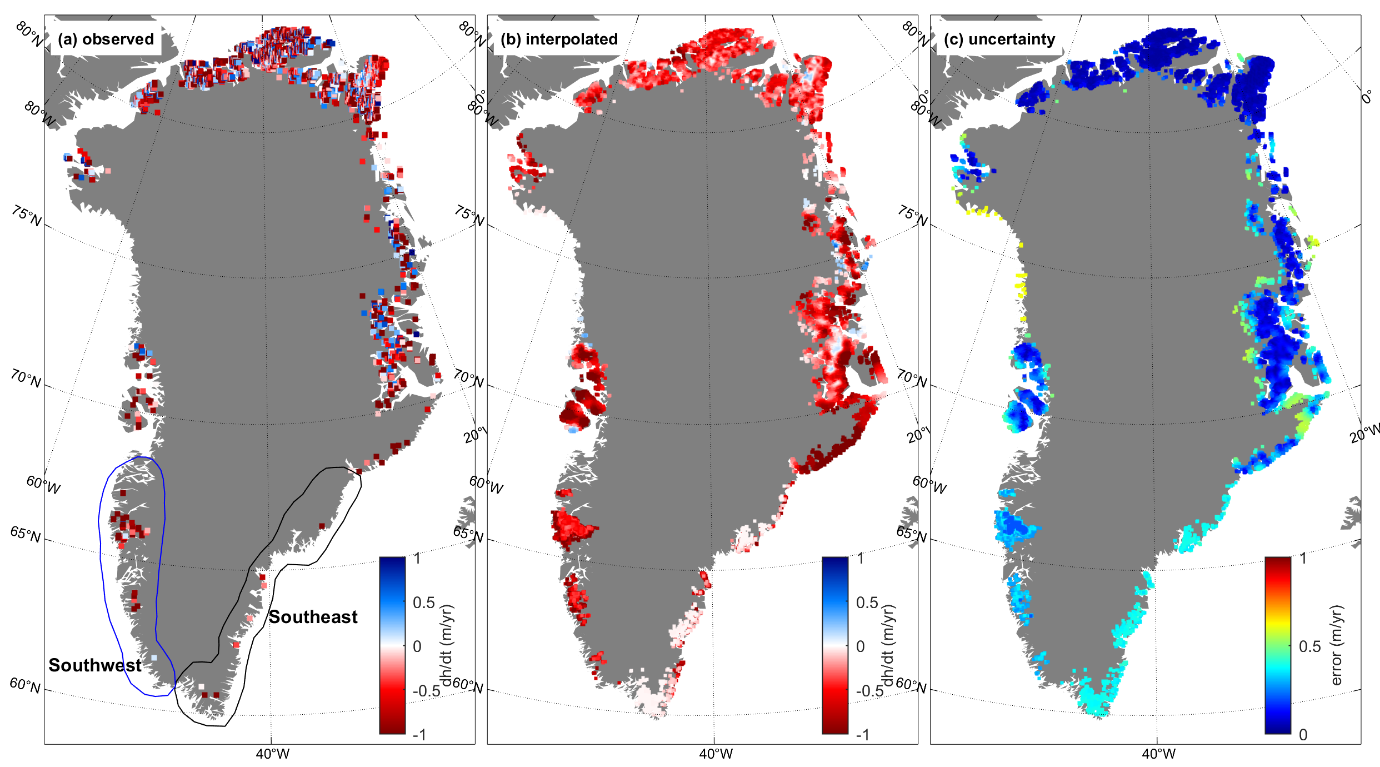
**

Figure S6. (a) Observed elevation change rates in m/yr during Oct 2008 – Apr 2019 using ICESat and UCESat-2 data. The black curve marks southeast Greenland and the blue curve marks southeast Greenland. (b) interpolated elevation change rates and (c) uncertainties of interpolated elevation change rates.

**
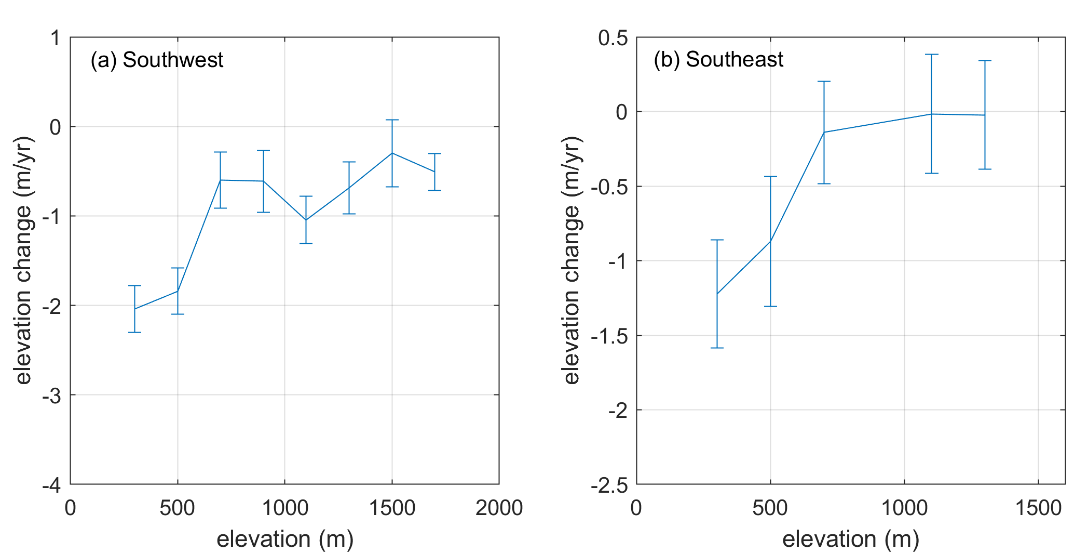
**

Figure S7. Elevation change rates (dh/dt) and standard deviations within 200 m elevation intervals for glaciers in (a) southwest Greenland and (b) southeast Greenland during Oct 2009 – Apr 2019 using ICESat and ICESat-2 data.

**Correction for glacial isostatic adjustment, elastic uplift, and firn compaction**

***Glacial Isostatic Adjustment***

The observed ice surface elevation changes were corrected for bedrock movement, caused by elastic uplift from present-day mass changes and long-term past ice changes (Glacial Isostatic Adjustment- GIA). To correct for GIA, we use the GNET-GIA empirical model of Khan et al. (2016) For each grid point on a 0.5x0.5 km grid, we estimate the GIA uplift rate dh_GIA_ and the associated uncertainty σ_GIA_.

***Elastic vertical land motion***

We correct for elastic uplift of the bedrock by convolving mass loss estimates (from ICESat, and ICESat-2) with the Green’s functions derived by Wang et al. (2012) for elastic Earth model iasp91 with refined crustal structure from Crust 2.0. For each grid point, we estimate the elastic uplift rate dh_elas_ and the associated uncertainty σ_elas._

***Converting volume to mass***

We convert volume to mass as described in Khan et al. (2014), however, with a few modifications. Conversion of the volume loss rate into the mass loss rate requires assumptions about density; using a constant ice density would be inaccurate. Firn compaction has to be taken into account to convert volume to mass correctly. Hence, elevation changes due to firn compaction are modeled with a simple firn model that includes melt and refreezing. It is forced by annual temperature, accumulation, melt, and refreezing from the regional climate model RACMO2.3p2 (Noël et al., 2018) at 5.5 km horizontal resolution. For every year, SMB is calculated as the sum of accumulation and refreezing minus melt, all with units [m ice yr^-1^]. The model assumes that all processes take place in the upper annual layer (i.e., that there is no deeper meltwater percolation). The surface layer contains a fraction of snow, and, where melt and refreezing occur, a fraction of refrozen ice.  The layer of refrozen ice that remains at the end of the melt season (hereafter referred to as *SIR* or Superimposed Ice Remaining) is equal to the refreezing from RACMO2.3p2 (Ligtenberg et al., 2018), albeit constrained between zero and SMB. The thickness and density of each annual layer is then the total of the two fractions, where the density of the firn layer at deposition is calculated by Reeh (2005).

$$\rho_{s0}=625+18.7 T+0.293 T^{2}$$

where *T­* is the firn temperature at 10m depth in °C, which depends on the mean annual temperature TMA and on SIR^14^,

$$T=TMA+26.6 SIR$$

As the surface layer is covered by subsequent layers firn compaction occurs, and hence the density of the firn fraction will increase and the thickness of the layer will decrease. The thickness of the annual layer *D*, which is deposited at time *t_0_*, after *t* years of compaction, will be:

$$D(t_{0},t)=SMB(t_{0})\frac{\rho_{i}}{\rho_{s0}\left( t_{0},t \right)}+SIR(t_{0})(1-\frac{\rho_{i}}{\rho_{s0}(t_{0},t)})$$

where *ρ_i_* is the density of ice (917 kg m^-3^), and *ρ_w_(t_0_,t)* is the density of the firn fraction of layer *D* after *t* years of compaction. To model the firn compaction process, the parameterization by Zwally and Li (2002) was used:

$$\rho_{s}\left( t_{0},t \right)=\rho_{i}-\left( \rho_{i}-\rho_{s0}(t_{0}) \right)e^{-ct}$$

where we use *c* from *Heron and Langway* (1980) given by:

$c_{0}=11exp\left( \frac{-10.16}{R T} \right)\dot{b}$ for $\rho\leq550$ kg/km^3^

$c_{1}=575 exp\left( \frac{-21.4}{R T} \right)\sqrt{\dot{b}}$ for $\rho>550$ kg/km^3^

where *R*=8.314 J mol^-1^ K^-1^ and mass accumulation rate $\dot{b}$.

The elevation change induced by *SMB* is taken as the anomaly of the thickness of the surface layer *D(t_0_)* with respect to the reference surface layer thickness (the 1961 to 1990 average). The total firn profile thickness at a given time is calculated as the sum of the thickness of 100 annual layers (not the surface layer) that each have compacted for the appropriate amount of time. For example, the profile thickness *D_T_* in 1985 is calculated as:

$$D_{T}\left( 2020 \right)=D\left( 1958,62 \right)+D\left( 1959,61 \right)+\ldots+D\left( 2019,1 \right)+\sum_{i=63}^{100} D(ref,i)$$

Because the RACMO2.3p2 run started in 1958, the upper 62 layers were modelled, and the profile was completed using the lower 37 layers from the reference profile (*D(ref,i);* 1961-1990 average profile). The largest firn compaction rates will occur in the upper layers, therefore the error introduced by assuming a partial reference profile will be minor. As more modeled layers become available later in the period of interest, this initialization error decreases. When the ice sheet is in balance, firn compaction does not cause a net surface elevation change. Thus, the elevation change due to firn compaction is again the anomaly of the profile thickness with respect to the 1961-1990 average (i.e., the total thickness of the reference profile).

We sum elevation changes due to firn compaction over the periods February 2003 to October 2009, October 2008 to April 2019, and October 2018 to December 2021. Table S1 shows our estimates of firn compaction rates in km^3^ yr^-1^. Once firn correction has been applied, we use the density of 917 kg/m3 to convert volume to mass.

We estimate firn compaction uncertainties as described in Kuipers Munneke et al. (2015) (see their equations 8 and 9) using input fields from RACMO2.3p2. For each grid point, we estimate the firn compaction rate dh_i,firn_ and the associated uncertainty σ_i,firn._ The total elevation change rate is for each grid point *i* is,

dh_i_ = dh_i,obs_ - dh_i,elas -_ dh_i,GIA –_ dh_i,firn_

The uncertainty is

$$\sigma_{i}=\sqrt{\sigma_{obs}^{2}+\sigma_{elas}^{2}+\sigma_{GIA}^{2}+\sigma_{firn}^{2}}$$

Table S1: corrections for firn compaction in Gt/yr.

| Region | Firn compaction rate  [Gt/yr] | Firn compaction rate   [Gt/yr] | Firn compaction rate   [Gt/yr] |
| --- | --- | --- | --- |
|  | Feb 2003 - Oct 2009 | Oct 2008 - Apr 2019 | Oct 2019 – Dec 2021 |
| North | -0.7 ± 0.2 | -1.0 ± 0.2 | -1.5 ± 0.2 |
| Northeast | -0.3 ± 0.1 | -0.2 ± 0.1 | -0.2 ± 0.1 |
| Southeast | 0.0 ± 0.1 | 0.0 ± 0.1 | 0.0 ± 0.1 |
| Southwest | -0.1 ± 0.1 | -0.2 ± 0.1 | -0.1 ± 0.1 |
| Northwest | -0.1 ± 0.1 | -0.1 ± 0.1 | -0.1 ± 0.1 |
| **All** | **-1.2 ± 0.3** | **-1.5 ± 0.3** | **-1.9 ± 0.3** |

Table S2: correction for elastic uplift due to present-day ice mass loss in Gt/yr.

| Region | Elastic correction  [Gt/yr] | Elastic correction   [Gt/yr] | Elastic correction   [Gt/yr] |
| --- | --- | --- | --- |
|  | Feb 2003 - Oct 2009 | Oct 2008 - Apr 2019 | Oct 2019 – Dec 2021 |
| North | 0.2 ± 0.1 | 0.3 ± 0.1 | 0.6 ± 0.1 |
| Northeast | 0.2 ± 0.1 | 0.2 ± 0.1 | 0.1 ± 0.1 |
| Southeast | 0.2 ± 0.1 | 0.2 ± 0.1 | 0.2 ± 0.1 |
| Southwest | 0.1 ± 0.1 | 0.1 ± 0.1 | 0.1 ± 0.1 |
| Northwest | 0.1 ± 0.1 | 0.1 ± 0.1 | 0.1 ± 0.1 |
| **All** | **0.8 ± 0.2** | **0.9 ± 0.2** | **1.1 ± 0.2** |

Table S3: Correction for GIA in Gt/yr (Khan et al., 2016).

| Region | GIA correction  [Gt/yr] |
| --- | --- |
|  | Feb 2003 - Dec 2021 |
| North | 0.1 ± 0.1 |
| Northeast | 0.1 ± 0.1 |
| Southeast | 0.0 ± 0.1 |
| Southwest | 0.0 ± 0.1 |
| Northwest | 0.0 ± 0.1 |
| **All** | **0.2 ± 0.1** |
